# Comparative Drug Release Investigations for Diclofenac Sodium Drug (DS) by Chitosan-Based Grafted and Crosslinked Copolymers

**DOI:** 10.3390/ma15072404

**Published:** 2022-03-24

**Authors:** Lalita Chopra, Kamal Kishor Thakur, Jasgurpreet Singh Chohan, Shubham Sharma, R. A. Ilyas, M. R. M. Asyraf, S. Z. S. Zakaria

**Affiliations:** 1 Department of Chemistry, University Institute of Sciences (UIS), Chandigarh University, Gharuan, Mohali 140413, Punjab, India; lalita.chemistry@cumail.in (L.C.); kamal.e8281@cumail.in (K.K.T.); 2 Mechanical Engineering Department, University Centre for Research & Development, Chandigarh University, Mohali 140413, Punjab, India; jaskhera@gmail.com; 3 Department of Mechanical Engineering, IK Gujral Punjab Technical University Main Campus, Kapurthala 144603, Punjab, India; 4 School of Chemical and Energy Engineering, Faculty of Engineering, Universiti Teknologi Malaysia, Johor Bahru 81310, Johor, Malaysia; ahmadilyas@utm.my; 5 Centre for Advanced Composite Materials, Universiti Teknologi Malaysia, Johor Bahru 81310, Johor, Malaysia; 6 Institute of Energy Infrastructure, Universiti Tenaga Nasional, Jalan IKRAM-UNITEN, Kajang 43000, Selangor, Malaysia; asyrafriz96@gmail.com; 7 Research Centre for Environment, Economic and Social Sustainability (KASES), Institute for Environment and Development (LESTARI), Universiti Kebangsaan Malaysia (UKM), Bangi 43600, Selangor, Malaysia

**Keywords:** pH-responsive, sustainable drug release, equilibration process, Fick’s law, diffusion

## Abstract

The hydrogels responding to pH synthesized by graft copolymerization only and then concurrent grafting and crosslinking of monomer *N*-isopropyl acrylamide (NIPAAM) and binary comonomers acrylamide, acrylic acid and acrylonitrile (AAm, AA and AN) onto chitosan support were explored for the percent upload and release study for anti-inflammatory diclofenac sodium drug (DS), w.r.t. time and pH. Diclofenac sodium DS was seized in polymeric matrices by the equilibration process. The crosslinked-graft copolymers showed the highest percent uptake than graft copolymers (without crosslinker) and chitosan itself. The sustainable release of the loaded drug was studied with respect to time at pH 2.2, 7.0, 7.4 and 9.4. Among graft copolymers (without crosslinking), Chit-*g*-polymer (NIPAAM-*co*-AA) and Chit-*g*-polymer (NIPAAM-*co*-AN) exhibited worthy results for sustainable drug deliverance, whereas Crosslink-Chit-*g*-polymer (NIPAAM-*co*-AA) and Crosslink-Chit-*g*-polymer (NIPAAM-*co*-AAm) presented the best results for controlled/sustained release of diclofenac sodium DS with 93.86 % and 96.30 % percent release, respectively, in 6 h contact time. Therefore, the grafted and the crosslinked graft copolymers of the chitosan showed excellent delivery devices for the DS with sustainable/prolonged release in response to pH. Drug release kinetics was studied using Fick’s law. The kinetic study revealed that polymeric matrices showed the value of *n* as *n* > 1.0, hence drug release took place by non-Fickian diffusion. Hence, the present novel findings showed the multidirectional drug release rate. The morphological changes due to interwoven network structure of the crosslinked are evident by the Scanning electron microscopy (SEM) analysis.

## 1. Introduction

Controlled and sustainable drug release offers one of the prime local medicinal chemistry. The target-oriented drug delivery devices carry and release the drug at a specific rate as per the body requirement for a definite period and also on an exact target [1,2]. The literature survey revealed that chitosan-based hydrogels were employed in the stimulus-targeted release of many drugs such as diclofenac sodium DS [3], 5-fluro-uracil (FU) [4], ketoprofen [5], ibuprofen [6], tetracycline [7], etc. V. L. Gonçalves at al. synthesized chitosan microspheres and investigated the liberation of DS drug from these polymeric matrices at 2.0, 6.8 and 9.0 pH under an in-vitro digestive tract environment [8]. Chitosan particles coated with poly (methyl acrylates) were observed as designed for the colon-selected release of the drug from the nanoparticles concerning time in simulated gastrointestinal fluids at a pH of 5.0 and 6.8 [9]. Chitosan grafted with binary monomers, glycidyl methacrylate (GMA) as monomer and acrylamide (Aam) and acrylonitrile (AN) as comonomers, GMA-*co*-AAm and GMA-*co*-AN were exhausted for the sustained release of drug at 7.4 pH. The grafted samples showed a very good result as proved to be excellent hydrogels as compared to pristine chitosan. V.H. Kulkarni [10] investigated crosslinked chitosan beads and microspheres, respectively, for encapsulation and sustainable release of DS with respect to time. Graft copolymerization or crosslinking of chitosan resulted in enhanced uploading of the drugs well as prolonged release with respect to time. Wang, Q. et al. in 2010 and 2012 reported acrylic acid)/vermiculite/sodium system graft copolymerized on chitosan backbone [11,12] for the equilibration of DS drug and its release inspired by gastric fluids of 2.1 pH and intestinal gut fluids with a pH of 6.8 and 7.4. Semi-Interpenetrating systems based on backbone chitosan and AAm-*g*-HEC were studied for the in vitro release of DS at 1.2 and 7.4 pH, respectively [13]. Pimwipha Piyakulawat and co-workers synthesized polyelectrolyte complex (PEC) hydrogel beads on chitosan and carrageenan (CR) support and used them as colon-specific delivery devices for the controlled release of diclofenac sodium (DFNa) drug stimulated by using in vitro gastrointestinal conditions [14]. When crosslinking was done by using glutaric acid and glutaraldehyde crosslinker, the beads obtained proved to be more efficient candidates for continued and prolonged release in 24 h for DFNa. The order of performance of beads was non-crosslinking beads < beads crosslinked with glutaric acid < beads Crosslink-linked with glutaraldehyde. With respect to pH, release was slower in pH 1.2 and 6.8 and quite high at pH 7.4. This finding showed that the release system is effective as a controlled release system for colon-specific drug delivery. In 2009, Li, Ning et al. coated liposomes onto low molecular weight chitosan (LCH) and utilized these as potential candidates for ocular delivery of diclofenac sodium as liposomes showed a prolonged drug release profile after coating [15]. V.G.M. Naidu et al. in 2009 also prepared Polyelectrolyte complexes (PEC) by using chitosan and gum kondagogu (GKG) via mixing polymeric solutions of different concentrations with respect to their weight to volume ratio. The product was then loaded with the DFNa drug. The drug release profile showed PEC presented a higher release of DFNa at pH 6.8 [16]. Wang and co-workers, fabricated a series of pH-sensitive beads of chitosan-*g*-poly(acrylic acid)/attapulgite/sodium alginate (CTS-*g*-PAA/APT/SA) crosslinked via Ca(II) ions and employed for cumulative release of diclofenac sodium (DS) with respect to pH. The drug release profile showed that drug release in the solution of pH 7.4 efficiently reached 100.0% only in 2.0 h contact time [17]. El-leithy et al. formulated hydrogels of various hydroxypropyl methylcellulose (different *w*/*w* ratio) and Carbopol 934 (1% *w*/*w*) consisting of DFS loaded CS microspheres. The fabricated mucoadhesive hydrogel formulations were investigated for the in vitro release of DFS and showed sustainable drug release reaching 34.60–39.70% after 6 h via a zero-order drug diffusion mechanism [18]. In 2019 Sun, Xiaoxiao et al. synthesized magnetism and pH-responsive microbeads composites of Fe_3_O_4_@C/carboxymethyl cellulose (CMC)/chitosan for in-vitro sustainable release of DS drug. The microbeads presented an advanced swelling index at pH 7.4 and 6.8 as compared to pH 1.2. The composite microbeads do not burst out at once and showed excellent in vitro drug release profiles in the gastrointestinal medium [19]. Sheikh Saudi et al. in 2020 employed electrospun nanofiber (EN) technology to prepare multi-layered-nanofibers that are electrostatically charged with Chitosan and polycaprolactone. The nanofibers were exploited for uploading and then prolonged-release diclofenac Sodium (DS) drug. The nanofibers presented efficient drug adsorption proficiency. Gradual drug-release profile was observed by the samples and at 336 h cumulative release by some of the samples was approximately 75.48% [20]. Nafisa Gull and her fellow co-workers synthesized hydrogels films by using chitosan and polyvinyl pyrrolidone crosslinked with epichlorohydrin for inflammation targeted controlled release of DS drug. The hydrogel films showed a highly efficient drug release profile of 87.56%o in contact time 130 min along with 84% drug encapsulation efficiency [21]. In March 2021, Ahmed M. Omer et al. published a research article based on encapsulation and deliverance of DS drug by pH-responsive microcapsules derived by using alginate (Alg) aminated chitosan (AmCs) and carboxymethyl chitosan (CMCs). The samples showed high DS release at pH 7.4 [22].

In 2014 authors investigated graft copolymerized GMA and comonomers onto chitosan support for controlled in vitro release of diclofenac sodium drug. Drug diffusion at various pHs was analyzed using Fick’s Law. Binary graft copolymers showed excellent results for sustained release of drug at 7.4 pH [23].

In the present work, already grafted copolymers and interlinked grafted copolymers of chitosan support synthesized by grafting and crosslinking of chitosan with monomer NIPAAM and comonomers acrylamide (AAm), acrylic acid (AA) and acrylonitrile (AN) [24,25] were examined for the disciplined deliverance of gastrointestinal non-steroidal anti-inflammatory drugs (NASID) diclofenac sodium (DS). DS drug was first equilibrated/uploaded in the graft copolymeric matrices. The drug-loaded matrices were then exhausted for the drug release at pH 2.2, 7.0, 7.4 and 9.4 with respect to time. The diffusion kinetics of drugs from the polymeric matrices was studied by Fick’s law.

In the earlier work, chitosan was grafted with NIPAAM by using initiator AIBN and optimal reaction parameters were evaluated. Dupal vinyl comonomers AAm, AA and AN were graft copolymerized with NIPAAM using the already reported optimal reaction parameters of NIPAAM itself only and optimal dupal vinyl comonomer’s concentration [24]. Crosslinked-graft copolymers were manufactured in the presence of *N*,*N*′-MBA at the optimal reaction parameters of grafting of NIPAAM only onto chitosan. At an optimum concentration of *N*,*N*′-MBA crosslinked binary graft copolymers were synthesized by using binary comonomer AAc, AAm and AN along with NIPAAM [25].

Pristine chitosan, graft copolymers, as well as interlinked grafted copolymers, were analyzed for the various morphological, structural and chemical changes instigated by grafting and crosslinking by diverse characterization practices such as TGA/DTA, FTIR, SEM and XRD. The swelling studies were also investigated at different pHs with respect to time to check their employability in enrichment and separation practices.

## 2. Materials and Methods

### 2.1. Materials Required

Chitosan, graft copolymers and interlinked grafted copolymers of chitosan support with monomer NIPAAM and comonomers AAm, AA and AN synthesized as discussed in earlier research publications [24,25], DS (Sigma Aldrich, Merck Ltd., Bangalore, India), NaOH, KH_2_PO_4_, KCl, concentrated HCl and Borax (from SD fine, Mumbai, India) were utilized as collected. All the above chemicals employed are of analytical rating.

### 2.2. Methodology

#### 2.2.1. Synthesis

Synthesis of graft copolymers and crosslinked graft copolymers of NIPAM onto chitosan was discussed in detail in the previous paper [24,25].

#### 2.2.2. Analysis

The changes in the structure and morphology of graft copolymers and interlinked grafted polymers were already discussed and proved by characterization techniques such as TGA/DTA (Hitachi STA7200 Thermal Analyzer; simultaneous TG/DT model, Hitachi Fukuoka, Japan), FTIR (Thermo Nicolet (Model 6700) spectrometer, Thermo Electron Scientific Instruments Corporation, Madison, WI, USA), XRD (X’ Pert PRO (PAN analytical, The Netherlands), SEM (JEOL India Pvt. Ltd., New Delhi, India), and swelling [24,25].

#### 2.2.3. Drug Adsorption on the Polymeric Matrices

**Figure 1:**
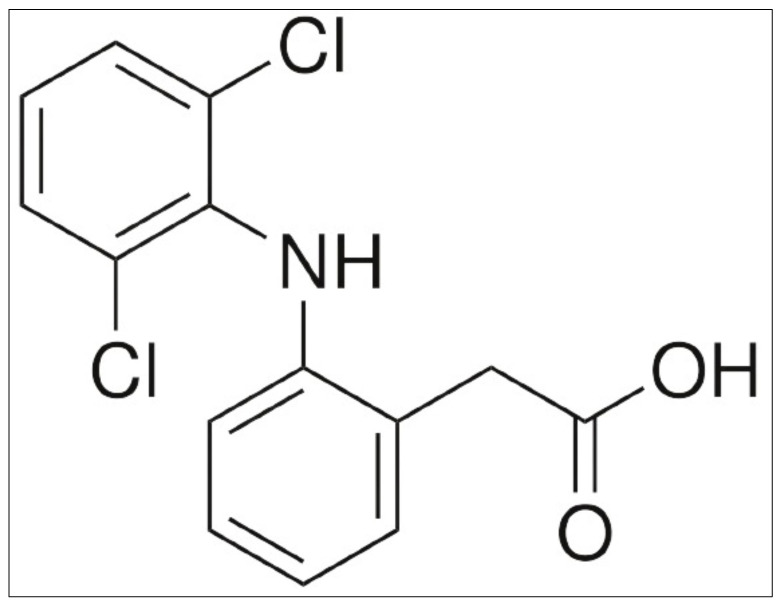
Diclofenac sodium (DS) Drug.

The equilibration technique was employed for the adsorption of drug DS onto chitosan and chitosan-supported graft copolymers and crosslinked-graft copolymers from the solution having 100 μg/mL of DS concentration [26]. The equilibration method is utilized for the adsorption of DS drug into polymeric matrices as depicted in Figure 1. Polymeric samples were kept immersed in the drug solution for 24 h for maximum uploading of the drug. The chitosan, graft copolymers and crosslinked-graft copolymers showing the highest percent grafting (P_g_) were investigated for the emancipation behavior of DS drug at diverse pH with respect to time. All evaluations of the DS solution were recorded at λ_max_ = 276 nm in a UV-visible spectrophotometer [27] (Thermo Evolution 300 model, Thermo Fisher Scientific Inc., Madison, WI, USA). A standard plot of drug solution was produced via plotting a graph amid drug absorption data and drug concentration as the drug concentration varied from 2 µg/mL to 100 µg/mL. A total of 25.00 mg polymeric samples were immersed in the 10.00 mL volume solution of DS having a concentration of 100 µg/mL for 24 h. Drug uploading in the polymeric samples was computed by recording absorption values of the filtrates.

Percent Drug uptake was calculated can be expressed as:

$$\mathrm{Percent}\;\mathrm{Drug}\;\mathrm{Uptake}\;=\;\frac{\mathrm{Total}\;\mathrm{drug}\;\mathrm{taken}\;\mathrm{in}\;\mathrm{solution}-\mathrm{Drug}\;\mathrm{left}\;\mathrm{in}\;\mathrm{supernatant}}{\mathrm{Total}\;\mathrm{drug}\;\mathrm{taken}\;\mathrm{in}\;\mathrm{solution}}\;\times \;100$$

#### 2.2.4. Drug Release

Samples (pristine chitosan, graft copolymers and crosslinked-graft copolymers) loaded with the drug DS were dipped in a 10.00 mL buffer solution of 2.2, 7.0, 7.4 and 9.4 pH to observe the release behavior following regular time intervals.

The percent drug releases by the polymeric samples with respect to time was calculated can be expressed as:

$$\mathrm{Percent}\;\mathrm{Drug}\;\mathrm{Release}=\frac{\mathrm{Concentration}\;\mathrm{of}\;\mathrm{drug}\;\mathrm{in}\;\mathrm{solution}}{\mathrm{Total}\;\mathrm{drug}\;\mathrm{taken}\;\mathrm{in}\;\mathrm{solution}}\;\times \;100$$

**Figure 2:**
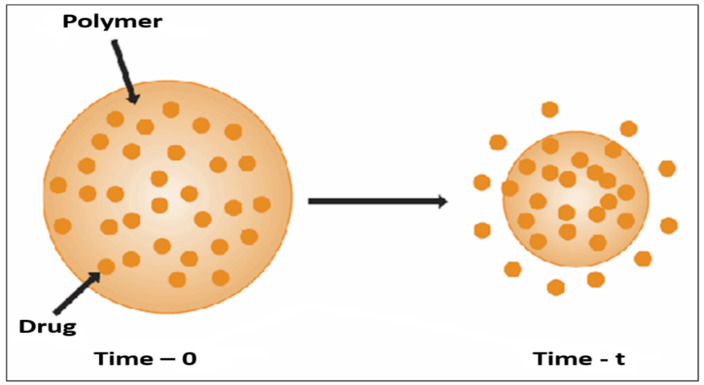
Diagrammatic representation of drug release with respect to time by the hydrogel.

The values for percent release of the drug were computed through equivalent absorption values of the drug solution from the standard curve as portrayed in Figure 2.

#### 2.2.5. Drug Release Kinetics

Korsmeyer-Peppas’s give Fick’s law to study the kinetics of drug diffusion [28] from chitosan and crosslinked-graft copolymers. The drug diffusion took place by Fickian or non-Fickian diffusion as illustrated in Table 1. According to Fick’s law, the portion of drug diffused at a particular period t was computed via the use of equation:

$$F=\frac{M_{t}}{M_{\infty }}=kt^{n}$$

In the above equation:

F—corresponds to fractional drug diffused out at period t,
M_t_—corresponds to drug diffused out at period t,
M_∞_—corresponds to drug diffused out at infinite period,
*n*—corresponds to diffusion exponent,
*k*—is gel characteristics constant,
t—is time in hours.

The *n* value is known through the slope of linear regression curve lotted amid $log\frac{M_{t}}{M_{\infty }}$ vs. log t. The numerical value of *k* is known through the intercept of the curve.

## 3. Results and Discussions

### 3.1. Comparative Analysis of Chitosan and Modified Polymeric Samples

Chitosan and the modified versions of the same were analyzed by the different characterization techniques [24,25] and were discussed already (FTIR, SEM, XRD, TGA/DTA and swelling studies). Physico-chemical changes appeared were compared with that of pristine chitosan, to get evidence of grafting.

#### SEM Analysis

**Figure 3:**
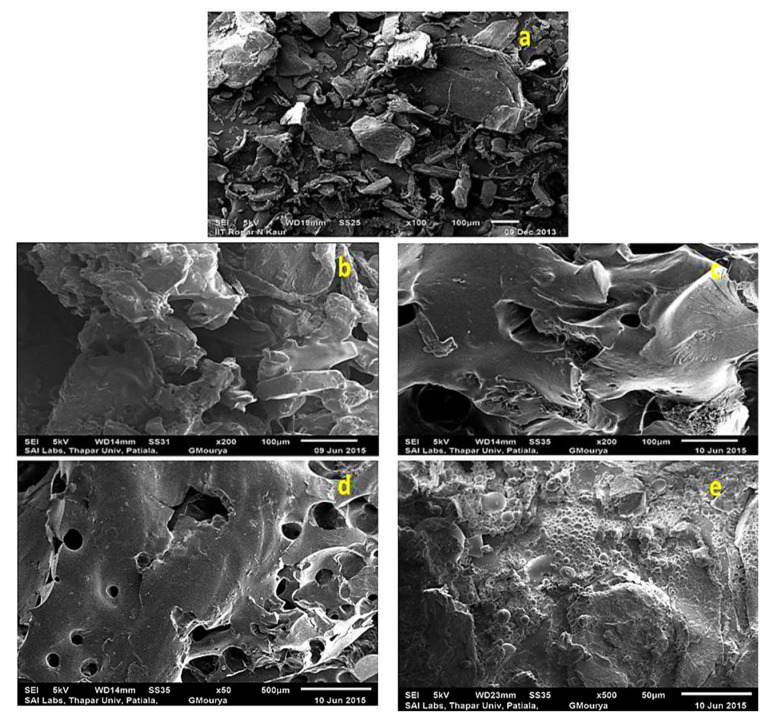
Comparative SEM analysis of chitosan backbone, graft copolymerized samples and crosslinked samples (**a**) chitosan, (**b**) Chit-*g*-polymer (NIPAAM) (**c**) Chit-*g*-polymer (NIPAAM-*co*-AA) (**d**) Chit-*g*-polymer (NIPAAM-*co*-AAm) (**e**) Chit-*g*-polymer (NIPAAM-*co*-AN).

**Figure 4:**
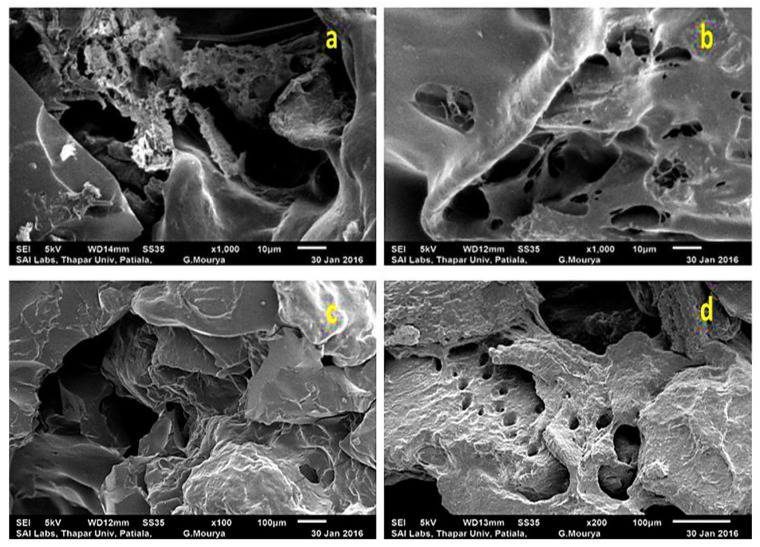
Comparative SEM analysis of chitosan backbone, graft copolymerized samples and crosslinked samples (**a**) Crosslink-Chit-*g*-polymer (NIPAAM) (**b**) Crosslink-Chit-*g*-polymer (NIPAAM-*co*-AA) (**c**) Crosslink-Chit-*g*-polymer (NIPAAM-*co*-AAm) (**d**) Crosslink-Chit-*g*-polymer (NIPAAM-*co*-AN).

The scanning electron microscope is a versatile, valuable, and well-accepted analysis technique for the reliable inspection of surfaces to know about surface morphology or to compare the surface morphology of unmodified samples with modified samples. In the present study, the scanning electron micrographs were recorded on JEOL India Pvt. Ltd., New Delhi, India. Pieces were incised from the sample films and were mounted onto stubs, dried, and coated with a gold coating. The polymeric samples were observed and photographs were taken under a scanning electron microscope with the build films Image Analyzing System at multiple magnifications, at an accelerating voltage of 15 kV. The SEM analysis at the low resolution was presented in this article as well to give a clear distinction of the conversion of rough crystalline flaky chitosan Figure 3a into soft spongy and porous structures (Figure 3b–e). With crosslinking, the porosity, sponginess and softness increased more (Figure 4a–d). The morphological changes due to the interwoven network structure of the crosslinked are visible in the SEM images.

### 3.2. Swelling Behavior

**Figure 5:**
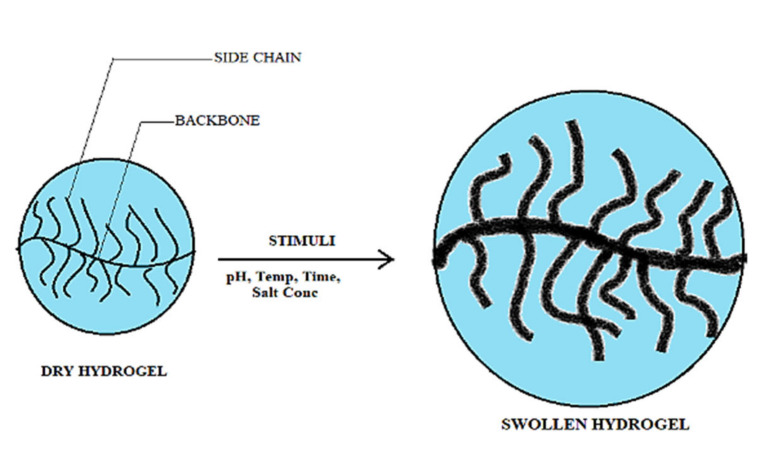
Swelling-deswelling of hydrogels with respect to stimuli pH, temperature, contact time, salt concentration.

Swelling is the key parameter to decide the drug uptake and also affects the release phenomenon by the hydrogels. Swelling and the deswelling behavior in response to the stimuli act as an important factor in the employability of these hydrogels in enrichment as well as separation technologies (Figure 5). The chitosan and the synthesized graft copolymers were investigated for the swelling studies already [24,25,26,27,28,29,30,31] and showed the highest swelling in the strongly alkaline medium at pH 9.4. Furthermore, grafting resulted in enhancement in the selling and crosslinking gave it more upsurge. Because of this the crosslinked grafted samples showed maximum swelling. Drug uptake and release depend on the type and magnitude of the interactions occurring among the systems as polymer and solvent; drug and polymer; and on the porosity of the sample (swelling observations of pristine chitosan, its grafted copolymers, and crosslinked matrices were shown and discussed in the earlier publication [17,18,29,30,31,32,33,34,35,36,37,38,39,40,41,42]). Maximum swelling was observed in a solution of pH 9.4, the order of effect of change in pH at swelling was concluded 9.4 > 7.4 > 7.0 > 2.2. This may be due to the fact that in basic medium cross-linked matrices experience reactions such as amide group hydrolysis, nitrile group hydrolysis, salt formation of the carboxyl group, etc. [43,44,45,46,47,48,49,50,51,52,53,54]. Crosslinked showed maximum P*_s_* in all mediums as compared to simple grafts and bare chitosan.

### 3.3. Drug Uploading and Release Study

#### 3.3.1. Comparative Drug Adsorption

**Figure 6:**
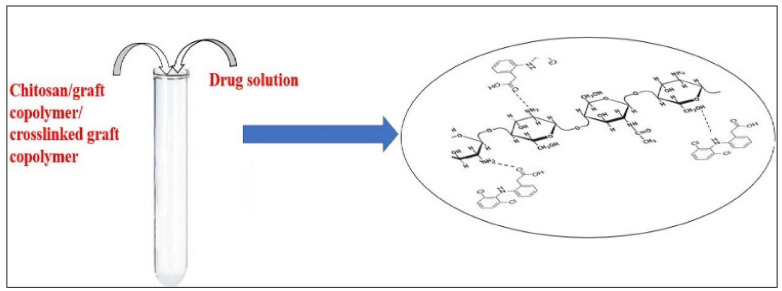
Drug-polymer interactions.

The percent drug uptake behavior of the chitosan and synthesized graft and crosslinked graft copolymers was shown in Table 1 and Figure 6, respectively. The percent drug uptake by all the graft copolymers was higher as compared to ungrafted chitosan.

The percent uptake was observed as 61.94% for Chit-*g*-polymer (NIPAAM), 87.13% for Chit-*g*-polymer (NIPAAM-*co*-AA), 82.30% for Chit-*g*-polymer (NIPAAM-*co*-AAm) and 75.79% for Chit-*g*-polymer (NIPAAM-*co*-AN). Chit-*g*-polymer (NIPAAM) and Chit-*g*-polymer (NIPAAM-*co*-AAm) get dissolved in the solution after three to four hours at all pH therefore further reading cannot be observed for these samples. The percent drug uptake by the crosslinked graft copolymers was high for the crosslinked graft copolymers as compared to the graft copolymers and chitosan. The percent uptake of 81.43%, 95.44%, 96.47% and 90.25% was observed for Crosslink-Chit-*g*-polymer (NIPAAM), Crosslink-Chit-*g*-polymer (NIPAAM-*co*-AAc), Crosslink-Chit-*g*-polymer (NIPAAM-*co*-AAm) and Crosslink-Chit-*g*-polymer (NIPAAM-*co*-AN) respectively as compared to chitosan 11.86%. This may be because these crosslinked graft copolymers swell more than another hence due to superior swelling drug emanates out from the crosslinked graft copolymers easily. The maximum swelling was shown by the Crosslink-Chit-*g*-polymer (NIPAAM-*co*-AAc) and Crosslink-Chit-*g*-polymer (NIPAAM-*co*-AAm) [25], which led to the opening up of polymeric network and resulted in the sustainable and controlled release of the DS as exhibited in Table 2.

#### 3.3.2. Comparative Drug Release Performance

**Figure 7:**
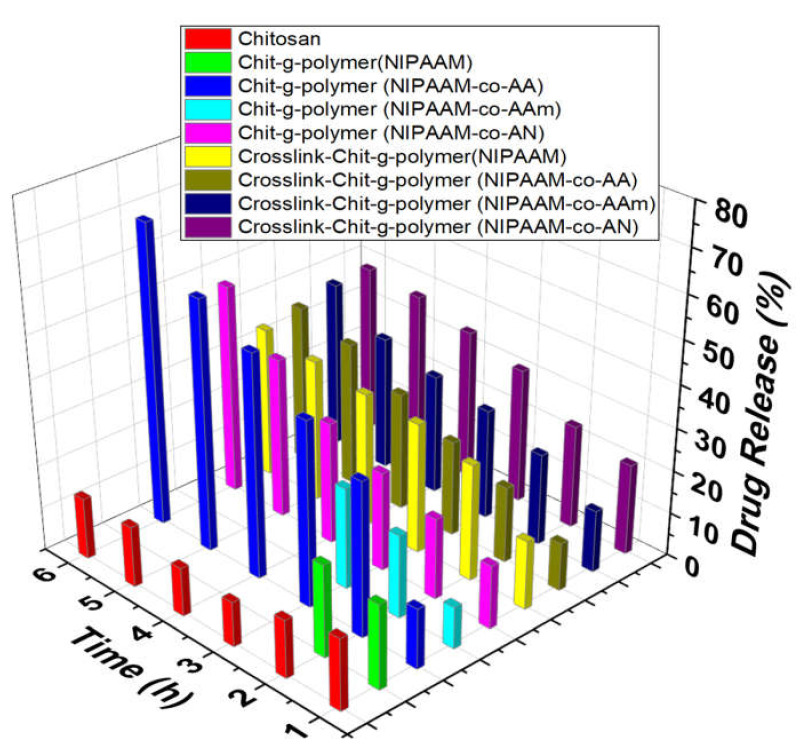
Drug Release performance of Grafted Copolymers and Crosslinked-graft Copolymers of NIPAAM and Binary Comonomers onto Chitosan. Polymeric taster = 25.00 mg, Concentration of DS solution for the drug-laden = 100.00 μg/mL, amount of buffer solution = 10.00 mL, pH of the solution = 2.2.

**Figure 8:**
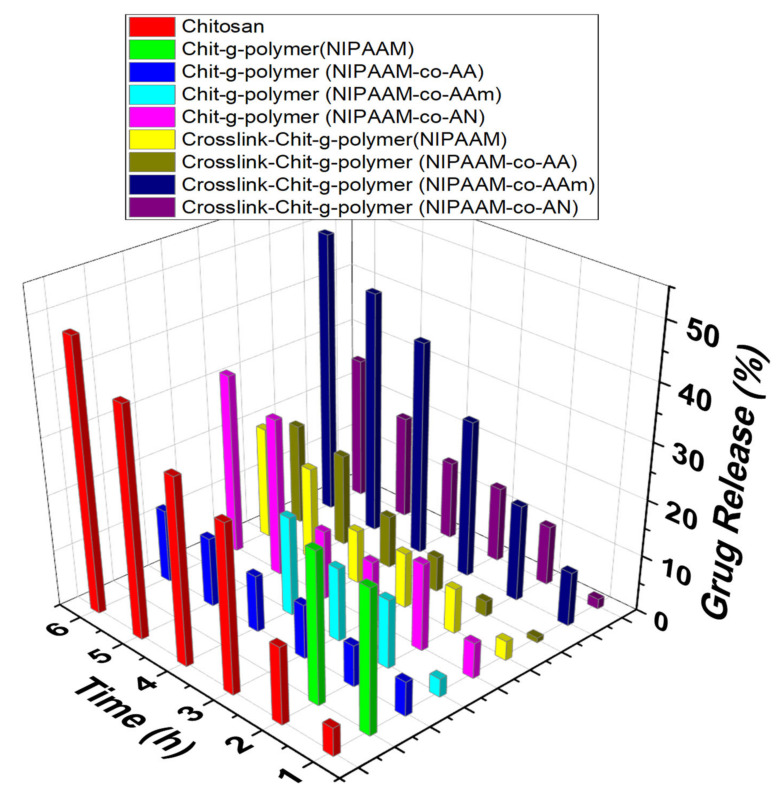
Drug Release performance of Grafted Copolymers and Crosslinked-graft Copolymers of NIPAAM and Binary Comonomers onto Chitosan. Polymeric taster = 25.00 mg, Concentration of DS solution for the drug laden = 100.00 μg/mL, amount of buffer solution = 10.00 mL, pH of the solution = 7.0.

**Figure 9:**
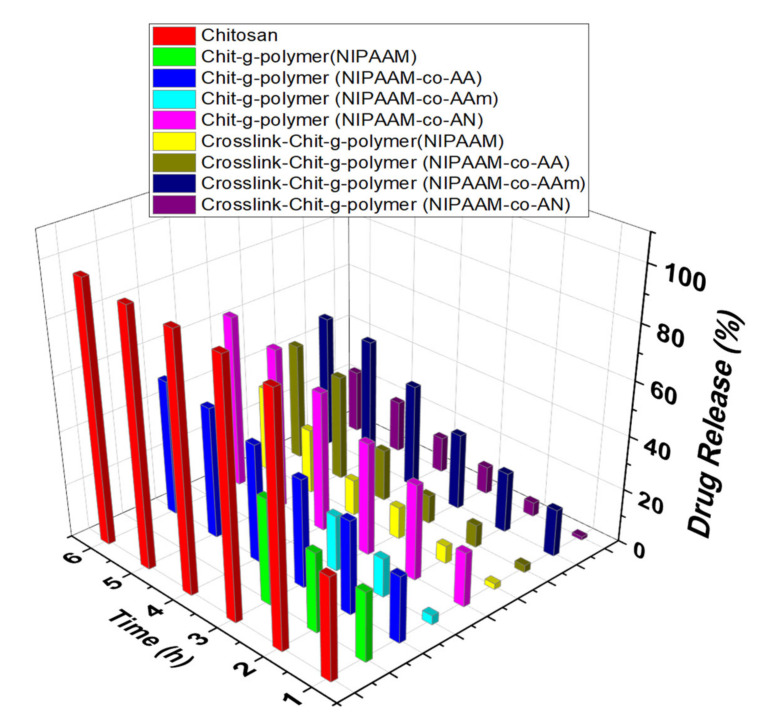
Drug Release performance of Grafted Copolymers and Crosslinked-graft Copolymers of NIPAAM and Binary Comonomers onto Chitosan. Polymeric taster = 25.00 mg, Concentration of DS solution for the drug-laden = 100.00 μg/mL, amount of buffer solution = 10.00 mL, pH of the solution = 7.4.

**Figure 10:**
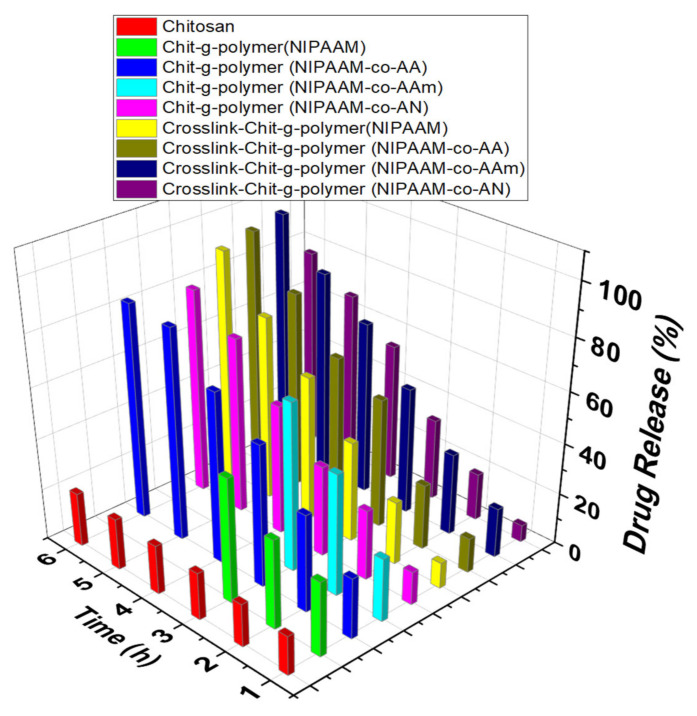
Drug Release performance of Grafted Copolymers and Crosslinked-graft Copolymers of NIPAAM and Binary Comonomers onto Chitosan. Polymeric taster = 25.00 mg, Concentration of DS solution for the drug-laden = 100.00 μg/mL, amount of buffer solution = 10.00 mL, pH of the solution = 9.4.

The drug release pattern revealed that maximum drug occurred up to 6 h contact hours and after that, there is no appreciable change in the drug release. In the case of graft copolymers, Chit-*g*-polymer (NIPAAM-*co*-AA) and Chit-*g*-polymer (NIPAAM-*co*-AAm) showed maximum uptake of 87.13% and 82.30%, respectively, as compared to 11.86% uptake by chitosan itself. At pH 2.2, the collective emancipation of DS through the chitosan is very low-slung after 6 h and there is no appreciable increase even up to 24 h. Whereas the comparatively high percent, drug release of 69.63% and 48.57% was shown by Chit-*g*-polymer (NIPAAM-*co*-AA) and Chit-*g*-polymer (NIPAAM-*co*-AN), respectively, in 6 h. When pH was 7.0 the percent drug release decreased and become even lesser than chitosan. Only 13.08% and 32.18% DS release was observed from Chit-*g*-polymer (NIPAAM-*co*-AA) and Chit-*g*-polymer (NIPAAM-*co*-AN) respectively 6 h time. With the increase in pH to 7.4 again a noticeable and sustainable DS release was observed with 50.72% and 64.47% drug release in 6 h by Chit-*g*-polymer (NIPAAM-*co*-AA) and Chit-*g*-polymer (NIPAAM-*co*-AN), respectively, but chitosan released more than 90.00% of the drug within 2 h. At pH 9.4, increased sustainable drug emancipation was recorded for the graft copolymeric samples with 92.31% and 92.32% drug release in 24 h by the Chit-*g*-polymer (NIPAAM-*co*-AA) and Chit-*g*-polymer (NIPAAM-*co*-AN), respectively, as compared to only 21.17% drug was released in 6 h by the pristine chitosan (Figure 7, Figure 8, Figure 9 and Figure 10).

In the case of crosslinked graft copolymers, a high percent uptake of 95.44% and 96.47% was observed for Crosslink-Chit-*g*-polymer (NIPAAM-*co*-AA) and Crosslink-Chit-*g*-polymer (NIPAAM-*co*-AAm), respectively, and other crosslinked-graft copolymers also exhibited a percent uptake of more than 80.00%. At pH 2.2, the cumulative drug release from the crosslinked-graft copolymers was very diminutive and a maximum of 38.64% and 38.99% drug release was shown by the Crosslink-Chit-*g*-polymer (NIPAAM-*co*-AAm) and Crosslink-Chit-*g*-polymer (NIPAAM-*co*-AN), respectively, in 6 h (Figure 7). At pH 7.0, there was a decrease in percent drug release by the crosslinked-graft copolymers except for Crosslink-Chit-*g*-polymer (NIPAAM-*co*-AAm) which showed a fringe increase in percent drug release (Figure 8).

At 7.4 pH the drug release percentage was added as related to the percent drug release at a pH of 2.2 and 7.0 and the best result was reported for Crosslink-Chit-*g*-polymer (NIPAAM-*co*-AAm) with a percent release of 49.77% (Figure 9). When pH is 9.4, the rate of release of DS was enhanced significantly for all the crosslinked-graft copolymers. This may be because these crosslinked-graft copolymers swelled better at pH 9.4 than another pH. The maximum swelling was shown by the Crosslink-Chit-*g*-polymer (NIPAAM-*co*-AA) and Crosslink-Chit-*g*-polymer (NIPAAM-*co*-AAm) at 9.4 pH, which resulted in the opening up of the polymeric network and resulted in the justifiable and intended release of the DS. Crosslink-Chit-*g*-polymer (NIPAAM-*co*-AA) and Crosslink-Chit-*g*-polymer (NIPAAM-*co*-AAm) presented superior outcomes for controllable/sustained release of drug with percent release of 93.86% and 96.30%, respectively, in 6 h contact time (Figure 10).

#### 3.3.3. Fick’s Diffusion Kinetics

**Figure 11:**
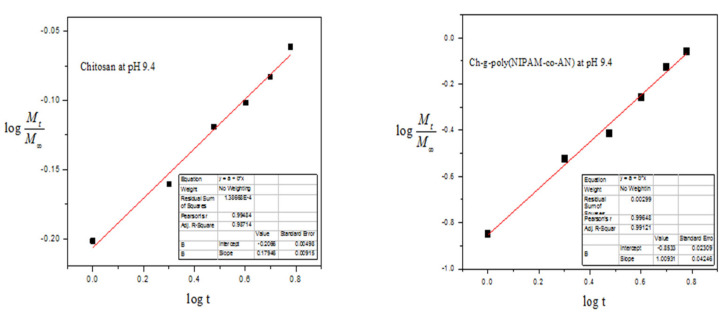
Release of DS kinetic study of Chitosan backbone and Chit-*g*-polymer (NIPAAM-*co*-AN) at pH 9.4.

Drug release kinetic studies of pure chitosan and grafted copolymers of NIPAAM and comonomers onto chitosan was shown in Figure 11.

Chit-*g*-polymer (NIPAAM) and Chit-*g*-polymer (NIPAAM-*co*-AAm) got dissolved in the solutions after two to three hours, therefore a kinetic study was not performed for these grafted copolymers. Chit-*g*-polymer (NIPAAM-*co*-AA) and Chit-*g*-polymer (NIPAAM-*co*-AN) showed best results at 9.4 pH for sustainable release of DS drug. For chitosan, the numerical *n* lies underneath 0.5 and specifies that drug release from chitosan occurred by Fickian diffusion. Chit-*g*-polymer (NIPAAM-*co*-AA) have *n* > 0.5 showed that drug release through the polymer matrices was owing to the non-Fickian type of diffusion. Chit-*g*-polymer (NIPAAM-*co*-AN) have a value of *n* > 1.0, hence drug release took place by non-Fickian diffusion super case-II (Figure 11).

**Figure 12:**
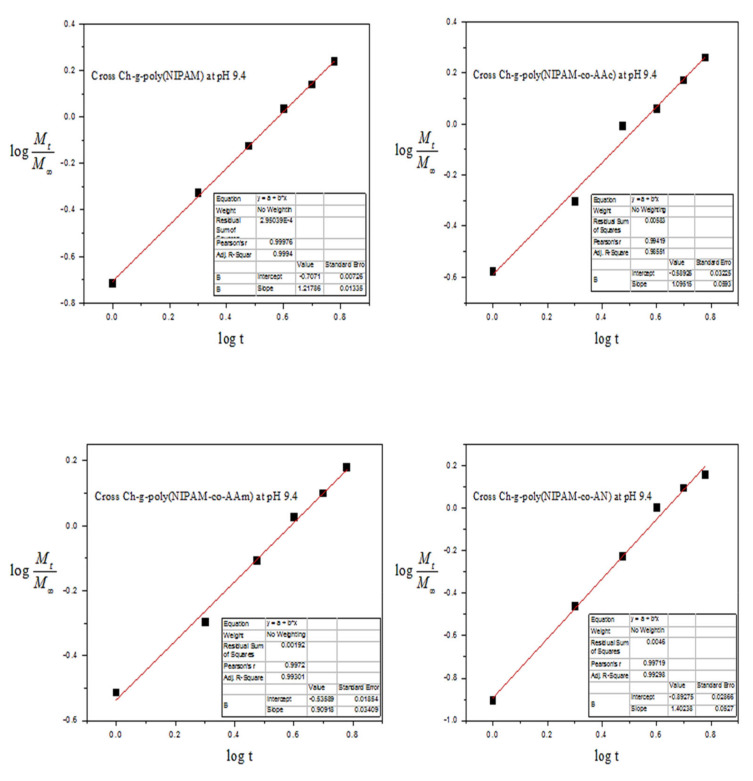
Drug release kinetics of Crosslink-Chit-*g*-polymer (NIPAAM), Crosslink-Chit-*g*-polymer (NIPAAM-*co*-AA), Crosslink-Chit-*g*-polymer (NIPAAM-*co*-AAm) and Crosslink-Chit-*g*-polymer (NIPAAM-*co*-AN) at pH 9.4.

Drug release kinetic studies of chitosan-supported crosslinked-graft copolymers with NIPAAM and comonomers are presented in Table 3 and Figure 12. Crosslink-Chit-*g*-polymer (NIPAAM-*co*-AAm) has a numerical value of *n* > 0.5, therefore DS drug diffused out from the polymeric sample owing to anomalous or non-Fickian diffusion. Crosslink-Chit-*g*-polymer (NIPAAM), Crosslink-Chit-*g*-polymer (NIPAAM-*co*-AA) and Crosslink-Chit-*g*-polymer (NIPAAM-*co*-AN) have value of *n* > 1.0, hence drug release took place by non-Fickian diffusion super case-II. The value of correlation coefficient ‘*r*’ approaches to unity supports the linear release of the drug.

## 4. Conclusions

Chitosan is explored as a hydrogel with a number of modifications such as derivatization, blends, meshes, nanoparticles, whiskers, etc. for delivery of a wide variety of drugs. Chitosan’s properties can be amended with requirements by the chemical modification through grafting and crosslinking. Crosslinking proved to be advantageous over simple grafting as it gives interwoven network gels with enhanced porosity. Novel pH-responsive graft copolymers and crosslinked-graft copolymers based on chitosan were investigated as promising applicants for the sustained/controllable release of DS an anti-inflammatory drug. As swelling is directly associated with drug uptake and its release, consequently it may be the primary factor that determines the enslavement of drug release onto pH and time. Grafting enhances the number of pores and sponginess resulting in higher sorption of the drug into the polymeric matrices. The percent drug uptake in chitosan was only 11.86% while graft copolymers showed very high percent uptake with best results of 87.13% by Chit-*g*-polymer (NIPAAM-*co*-AA). Crosslinking resulted in an upsurge in the numeral of pores, sponginess, and interlinking cavities in the polymeric samples resulting in higher loading of the drug molecules in the polymers. Therefore, the percent drug uptake was increased by grafting and crosslinking and ordered as crosslinked-graft copolymers > graft copolymers > chitosan. All the binary crosslinked graft copolymers showed drug uptake of more than 90.00% with Crosslink-Chit-*g*-polymer (NIPAAM-*co*-AAm) showing maximum uptake of 96.47%. The cumulative drug release under acidic (pH 2.2) and neutral (pH 7.0) environments was found to be comparatively less than in the alkaline medium (pH 7.4 and 9.4). At pH 9.4, appreciable sustainable drug release was reported with 92.31% and 92.32% drug release in 24 h by the Chit-*g*-polymer (NIPAAM-*co*-AA) and Chit-*g*-polymer (NIPAAM-*co*-AN), respectively, whereas crosslinked-grafted copolymers displayed best results for the sustainable release with higher than 93.00% drug release in 6 h time by Crosslink-Chit-*g*-polymer (NIPAAM-*co*-AA) and Crosslink-Chit-*g*-polymer (NIPAAM-*co*-AAm), respectively. The inference is drawn from the above experimentation that chitosan itself is a polymer of biological origin along with hydrogel properties. Yet there are some limitations due to the crystal flaky structure (shown in SEM Images) in terms of solubility. The horizon of its applications was not too broad as well as excellent. For the betterment of the required properties, it is chemically modified by graft copolymerization and then by crosslinking as well to study the impact of these two chemical changes on its applicability.

The altered chitosan (by grafting and crosslinking) resulted in better swelling in terms of time and sustainability. It therefore, showed better drug upload and sustainable release within 24 h. If the drug uptake is higher than it will impact release in a better way. The sustainable and prolonged release of the drug by the polymeric hydrogel increased its potential as a delivery vehicle for a prolonged time as it also showed the best release at pH 9.4 which means it release the drug by obtaining pH stimuli as 9.4 of the intestinal regions.

Therefore, it proved to be the best delivery vehicle of model drug DS (gastrointestinal drug).

## Figures and Tables

**Table 1:** Fickian and non-Fickian diffusion with respect to *n* values.

Value of *n*	Diffusion Type
≤0.5	Fickian diffusion
=0.5	The drug diffuses and releases out following the Fickian diffusion
>0.5	Anomalous or non-Fickian type drug diffusion
≥1	Comprehensively non-Fickian one or the Case II release kinetics is functioning

**Table 2:** Drug Uptake by Chitosan, graft copolymers and Crosslinked Graft Copolymers ^a^ of NIPAAM and Binary Comonomers onto Chitosan.

S. No.	Polymer	Temp.	pH	% Drug Loaded
1.	Chitosan	30 °C	7.0	11.86
2.	Chit-*g*-polymer(NIPAAM)	30 °C	7.0	61.94
3.	Chit-*g*-poly (NIPAAM-*co*-AA)	30 °C	7.0	87.13
4.	Chit-*g*-poly (NIPAAM-*co*-AAm)	30 °C	7.0	82.30
5.	Chit-*g*-poly (NIPAAM-*co*-AN)	30 °C	7.0	75.79
6.	Crosslink-Chit-*g*-polymer (NIPAAM)	30 °C	7.0	81.43
7.	Crosslink-Crosslink-Chit-*g*-polymer (NIPAAM-*co*-AA)	30 °C	7.0	95.44
8.	Crosslink-Chit-*g*-polymer (NIPAAM-*co*-AAm)	30 °C	7.0	96.47
9.	Crosslink-Chit-*g*-polymer (NIPAAM-*co*-AN)	30 °C	7.0	90.25

^a^ Polymer sample = 25.00 mg, Conc. of drug solution for drug loading = 100.00 μg/mL.

**Table 3:** DS Release Kinetic study of Grafted Copolymers of NIPAAM and Crosslinked-graft Copolymers ^a^ of NIPAAM and Binary Comonomers onto Chitosan.

Sr. No.	Polymeric Matrices	pH 9.4		
		*n*	*K*	*r*
1.	Chitosan	0.17946	0.6214	0.99484
2.	Chit-*g*-polymer(NIPAAM-*co*-AA)	0.74239	0.2561	0.99632
3.	Chit-*g*-polymer(NIPAAM-*co*-AN)	1.00931	0.1402	0.99648
4.	Crosslink-Chit-*g*-polymer(NIPAAM)	1.2179	0.1963	0.9998
5.	Crosslink-Chit-*g*-polymer (NIPAAM-*co*-AA)	1.0951	0.2527	0.9942
6.	Crosslink-Chit-*g*-polymer (NIPAAM-*co*-AAm)	0.9092	0.2911	0.9972
7.	Crosslink-Chit-*g*-polymer (NIPAAM-*co*-AN)	1.4024	0.1280	0.9972

^a^ Polymer sample = 25.00 mg, Conc. of drug solution for drug loading = 100.00 μg/mL.

## Data Availability

The data presented in this study are available on request from the corresponding author.

## Abbreviations

Chit	Chitosan
NIPAAM	*N*-isopropylacrylamide
AA	acrylic acid
Aam	acrylamide
DS	diclofenac sodium
-*g*-	graft
-*co*-	copolymer
Crosslink	crosslinked
GKG	gum kondagogu
*N*,*N*′-MBA	*N*,*N*′-Methylenebisacrylamide
FU	5-fluro-uracil
AIBN	Azobisisobutyronitrile
pH	power of hydrogen
PEC	polyelectrolyte complex
DFNA	diclofenac sodium
CST	Chitosan
LCH	low molecular weight chitosan
CR	carrageenan
PAA	poly(acrylic acid)
APT	attapulgite
SA	sodium alginate
EN	Electro spun nanofiber
CMC	carboxymethyl cellulose
GMA	Glycidyl methacrylate
Fe_3_O_4_	Ferrosoferric oxide
NSAID	Non-steroidal anti-inflammatory drugs
NaOH	Sodium hydroxide
KH_2_PO_4_	Potassium hydrogen phosphate
KCl	Potassium chloride
Conc. HCl	Concentrated Hydrochloric acid
